# Parallel photocycle kinetic model of anion channelrhodopsin *Gt*ACR1 function

**DOI:** 10.1016/j.bpj.2024.05.016

**Published:** 2024-05-18

**Authors:** Istvan Szundi, David S. Kliger

**Affiliations:** 1 Department of Chemistry & Biochemistry, University of California, Santa Cruz, Santa Cruz, California

## Abstract

The light-gated anion channelrhodopsin *Gt*ACR1 is an important optogenetic tool for neuronal silencing. Its photochemistry, including its photointermediates, is poorly understood. The current mechanistic view presumes BR-like kinetics and assigns the open channel to a blue-absorbing L intermediate. Based on time-resolved absorption and electrophysiological data, we recently proposed a red-absorbing spectral form for the open channel state. Here, we report the results of a comprehensive kinetic analysis of the spectroscopic data combined with channel current information. The time evolutions of the spectral forms derived from the spectroscopic data are inconsistent with the single chain mechanism and are analyzed within the concept of parallel photocycles. The spectral forms partitioned into conductive and nonconductive parallel cycles are assigned to intermediate states. Rejecting reversible connections between conductive and nonconductive channel states leads to kinetic schemes with two independent conductive states corresponding to the fast- and slow-decaying current components. The conductive cycle is discussed in terms of a single cycle and two parallel cycles. The reaction mechanisms and reaction rates for the wild-type protein, the A75E, and the low-conductance D234N and S97E protein variants are derived. The parallel cycles of channelrhodopsin kinetics, its relation to BR photocycle, and the role of the M intermediate in channel closure are discussed.

## Significance

The search for improved optogenetic tools will be enhanced by developing an understanding of the mechanisms by which anion and cation channels respond to light excitation. Most studies of these channels, including the *Gt*ACR1 anion channel, have assumed a photochemical mechanism parallel to the mechanism of bacteriorhodopsin (BR). Here, we show that this assumption is incorrect and determine characteristics of the photoreactions of *Gt*ACR1 and several of its variants. In the process we discuss factors affecting channel opening, show that this anion channel undergoes multiple parallel photocycles, and that, while the spectral characteristics of the intermediates of this channel mimic spectral characteristics of BR, the nature of the intermediates are unrelated to those of BR intermediates.

## Introduction

Naturally occurring anion channelrhodopsin *Gt*ACR1 is an efficient optogenetic tool used to control membrane potential through hyperpolarization during inhibition of neuronal firing (1,2,3). It has been the subject of a wide range of structural and functional investigations that resulted in establishing its crystal structure (4,5), the role of amino acids in its function (6,7), and a broad range of other properties reviewed in (8). Most of the investigations have been focusing on the functional role of amino acids at or near the protonated retinal Schiff base, and on the nature of its potential counterions, which are known to play important roles in bacteriorhodopsin (BR) function. The structure of the ion channel, especially the constriction points, is another highly investigated area that has the potential of making significant improvements by engineering superior ion channels. The detailed crystal structure available provides a rich background for these studies. Much less is known about the dynamics of the protein and its relation to the channel function. Time-dependent absorption spectroscopy based on the single wavelength measurement technique has been applied to the wild-type (WT) protein and its results were compared with the time-resolved current data published (6,9). Based on the results of the comparison, a photocycle mechanism was proposed. According to that mechanism, the K intermediate decays to the L-like intermediate on the μs timescale and the L-like intermediate itself is responsible for the open channel state. Channel closing occurs in two phases; the first phase involves the partial decay of the L-like intermediate and the temporary deprotonation of the Schiff base, forming the M intermediate in a reversible mechanism. In the second phase both the L-like and M intermediates get depleted irreversibly forming the N, and possibly the O, intermediates which decay during the slow recovery of the dark form on the minutes timescale. The design of the photocycle follows the steps in the BR photochemistry, considered to be the standard for the bacterial rhodopsin family. Recently, based on FTIR and visible spectroscopy results, an improved version of the mechanism was introduced (10), which suggested multiple forms of the L intermediate: nonconductive early ones in equilibrium with the K intermediate and having a 450 ns lifetime, and a conductive one emerging from them with an 18 *μ*s lifetime according to the IR data.

Very recently, time-resolved absorption spectroscopy, with detection in a broad spectral and time range, was applied to the WT protein, the A75E and two low-conductance mutants, D234N and S97E, at physiological and acidic pH (11). Exponential fitting and spectral analysis revealed four spectral forms and six sequential intermediates having composite spectra of the four spectral forms. It was concluded that the *Gt*ACR1 photocycle contains isospectral intermediates. The time evolutions of the spectral forms for the proteins studied were inconsistent with the existing photocycle model. It raised questions about the nature of the intermediate responsible for channel opening and the role of the Schiff base deprotonation in the channel kinetics. We made a direct comparison between the current kinetics and the time evolutions of the spectral forms and concluded that it is not the blue-absorbing L-like intermediate but an intermediate with a red-absorption spectrum that is responsible for the channel opening (12).

In this work we report on a comprehensive quantitative kinetic analysis of the *Gt*ACR1 photoreactions for the WT protein at acidic, physiological, and alkaline pH, for the A75E mutant at physiological pH, and for two low-conductance mutants, D234N and S97E, at physiological and acidic pH. We show that the kinetic mechanism involves parallel conductive and nonconductive photocycles. The intermediates in the cycles are identified and the reaction rates in the kinetic schemes for both cycles are derived. We argue that, while the BR kinetics is usually interpreted using single photocycle models, the photoreactions of the anion channelrhodopsin *Gt*ACR1, similarly to the cation channelrhodopsin-2 *Ps*CCR2 (13), require parallel photocycle models for quantitative description.

## Materials and methods

### Published data used in the analysis

The experimental data analyzed in this work were published previously (11). We reported time-resolved optical absorption spectra in the 350–700 nm wavelength range and 100 ns to 1 s time interval. The data were analyzed using singular value decomposition, global exponential fitting, and spectral deconvolution. Four independent spectral forms undergoing five exponential transitions were identified and their time evolutions were calculated. In this presentation we rely on the following results of our previous work: the apparent lifetimes and b-spectra from the exponential fit, the spectral forms derived from sequential intermediate spectra, the spectral composition of intermediate spectra (the composition matrix), and the time-dependent concentrations of the spectral forms. In the present analysis we also use published electrophysiological data (6,9,14).

### Scheme fitting

Kinetic matrices for degenerate and extended schemes are obtained by a fitting procedure, which adjusts the microscopic rates in the kinetic matrix to reproduce both the apparent rates and b-spectra of the global exponential fit. The reproduced b-spectra, ***bs***, are the product of the spectra of the presumed intermediates, ***E***, and the eigenvectors, ***W***, of the fitted kinetic matrix: ***bs = E***×***W***. The spectral forms derived in the deconvolution of the sequential intermediates serve as spectra of intermediates in the scheme. In case of degenerate schemes the eigenvectors belonging to the degenerate eigenvalues of the kinetic matrix are combined before calculating the b-spectra of the fit.

## Results and discussion

In our recent report (11) we presented the time-resolved optical absorption spectra of the *Gt*ACR1 photoreaction in the 350–700 nm wavelength and 100 ns to 1 s time intervals. Because this work relies heavily on the published results, a brief summary of them is given here. Singular value decomposition was consistent with four independent spectra, while exponential fitting gave five apparent rates and six amplitude b-spectra which were converted into six sequential intermediate spectra. In agreement with the singular value decomposition results, the spectral deconvolution of the sequential spectra yielded four independent spectral forms: a red-absorbing K-like, a blue-shifted L-like, a deprotonated M, and a recovered R form. It was concluded that the *Gt*ACR1 photoreactions must have isospectral intermediates. The four spectral forms derived had the characteristic shapes of the protein absorption spectrum when viewed on the energy (wavenumber) scale, and thus are accepted to represent the spectra of intermediates in the photocycle. We have also compared the channel currents recorded in electrophysiological experiments with the time evolutions of the K-like and L-like spectral forms (12). We found that the red-absorbing K-like spectral form always follows the current kinetics, while the blue-absorbing L-like form does not in most of the cases. We thus proposed the red absorbing intermediate for the open channel state. The time evolutions of the K-like and the L-like spectral forms were found to span the entire time range of the reaction. Assuming a single reaction chain, the intermediates represented by the two spectral forms should thus form equilibrated mixtures throughout the photocycle. The time-dependent concentration profiles of all four spectral forms, however, were not consistent with this concept (12). We argued that adequate description of the *Gt*ACR1 photocycle kinetics can be carried out successfully only within the concept of parallel photocycles.

In the kinetic analysis we derive the photocycles from the spectroscopic data in such a way that also makes the parallel cycles consistent with electrophysiology data. The current traces will be used to determine the amount of the K-like spectral form that belongs to the conductive cycle. We do not, however, make any attempt to obtain the best fit to the current signal shape because the current shape reflects more than the reaction kinetics alone, as discussed below. In the derivation of photocycle kinetics we will not follow the familiar trial-and-error statistical scheme-fitting technique. Instead, we use the less familiar algebraic method, described below in detail, which allows us to obtain the possible kinetic mechanisms by analyzing the spectral compositions of the sequential intermediates published in our first communication (11). A detailed and quantitative description of the *Gt*ACR1 kinetics is given below.

### Partitioning the spectral forms between conductive and nonconductive cycles and defining the intermediates in the cycles

The results of the spectroscopic experiment leave important questions unanswered. First, to what intermediate states the spectral forms should be assigned and, second, what are the roles those intermediates play in the photocycles. To answer these questions, we combine the spectroscopic results with the photocurrent data published for the WT and mutant proteins. Aligning the flash-induced channel current with the time evolution of the red-absorbing K-like spectral form defines unambiguously the portion of the K-like form that belongs to the conductive cycle and allows us to partition the spectral forms between two reaction chains and assign them to intermediates of the parallel photocycles.

**Figure 1:**
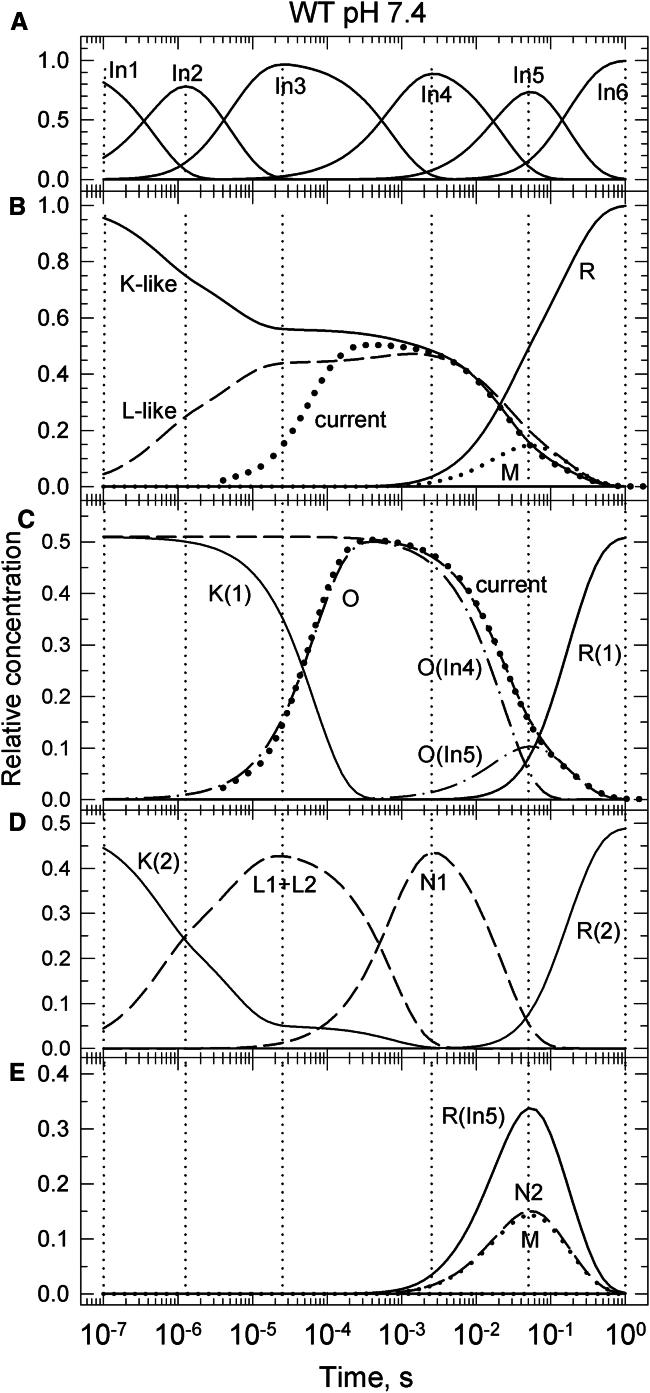
Partitioning the spectral forms between conductive and nonconductive parallel photocycles based on the channel current (6). (*A*) Time evolution of sequential intermediates derived from the exponential fit (*solid lines*). The dotted lines are drawn at their peak position. (*B*) Alignment of current (*dotted line*) with the K-like spectral form profile (*solid line*). (*C*) Time evolution of the K (*solid*), O (*dash-dot*), and R(1) (*solid*) states in the conductive Cycle1. The K-like spectral form assigned to Cycle1, dashed line, is split between the K and the conductive O states. The K→O step follows the current (*dotted*) onset kinetics. The O concentration is the sum of its fast-decaying O(In4) and slow-decaying O(In5) components, both dash-dot lines. (*D*) Time evolution of the intermediate states in the nonconductive Cycle2. (*E*) Same for the intermediate states shared between the conductive and nonconductive cycles.

A step-by-step procedure is demonstrated here using the spectral and current information obtained for the WT protein at pH 7.4. The time evolution of the six sequential intermediates, In1-In6, shown in Fig. 1 *A*, guides us in the sequence of events. The dotted vertical lines drawn at the peaks of the concentration profiles across the panels allow us to relate the events in the parallel cycles to the lifetimes and sequential intermediates obtained in the exponential fit. We have to emphasize that the sequential scheme is not the real mechanism of the *Gt*ACR1 kinetics; its intermediates are not molecular states but mixtures of them, as shown in the composition matrices (11). The scheme was introduced not as the mechanism but as a useful tool to serve analysis purposes. The real mechanism will emerge from this study, and its intermediates have spectra identical to the spectral forms. To avoid any confusion, the intermediates of the parallel photocycles will be called intermediate states, or more often just states.

Fig. 1*B* shows the time evolutions of the four spectral forms, the K-like, L-like, M, and R, derived from the sequential intermediates. The composition of the sequential intermediates in terms of spectral forms can be read following the dotted vertical lines drawn at the peaks of the concentration profiles in Fig. 1 *A*. More accurate values can be found in the 4 × 6 composition matrices published earlier (11). In1, In2, In3, and In4 contain only the K-like and L-like forms in different amounts. In5 has all four spectral forms and In6 is solely the recovered R form. In Fig. 1 *B* the current, matching the late part of the K-like spectral form evolution, is shown by a dotted line. The current rises in the first half of the time gap between In3 and In4, and its rate is somewhat faster than the third apparent rate that connects In3 to In4. The onset of the current was not detected spectrally, suggesting that the intermediate states before and after the channel opening have the same spectra. The sum of intermediate state concentrations before and after channel opening should be constant and should match the current plateau, which has a level of 0.51. Thus, the amount of the K-like spectral form that belongs to the conductive cycle is unambiguously determined by the channel current and its time dependence is shown in Fig. 1 *C* (*dashed line*). Following the BR nomenclature, explained in detail below, we call the state before channel opening the K state, labeled K(1), and the one corresponding to the open channel we call the O state not because it represents the open state but because it is a red-absorbing intermediate present in the ms time window as in BR kinetics, solid and dash-dot lines in Fig. 1 *C*, respectively. The transition between them occurs with the rise time of the current. These intermediate states constitute the backbone of the conductive cycle, Cycle1. Using a single K→O step for the first part of the conductive cycle may seem an oversimplification; however, no spectral trace was detected to support a more complex K decay path.

The amounts of the K-like spectral form thus assigned to the conductive cycle in the sequential intermediates are: 0.51 in In1-In3 (columns 1–3) named K(1), all 0.51 in In4 (column 4) named O(In4), and all 0.14 in In5 (column 5) named O(In5). The cycle also has 0.51 of R in In6 (column 6) named R(1). The partitioning and assignment of the L-like, M, and R spectral forms present in In5 (column 5) are not guided by the experimental data. These are called the shared spectral forms, and are not included in Fig. 1 *C* but are shown separately in Fig. 1 *E*. Any combination of them, totaling 0.37 fraction value, complements the conductive cycle. This results in multiple choices in the design of the cycle recovery steps, as discussed below. However, because these spectral forms in In5 are all the same type, nonconductive and cycle-closing states, the way they are partitioned between the cycles plays no significant role in the interpretation of the channel function.

The K-like spectral form assigned to Cycle1 is a 0.51 fraction of the total. The remaining 0.49 fraction should be assigned to a second cycle, which does not contribute to the channel conductance. We call it the nonconductive cycle, Cycle2. Removing the concentration profile of the K-like spectral form assigned to Cycle1, dashed line in Fig. 1 *C*, from the overall K-like trace in Fig. 1 *B*, results in the trace labeled K(2) in Fig. 1 *D*. Pairing it with the L-like trace, labeled L1 + L2 in Fig. 1 *D*, reveals the early steps of the photoreaction. The traces are fully consistent with a two-step equilibration between a K-like intermediate, called the K state, and two L-like intermediates called the L1 and L2 states. The intermediate mixture produced in the sub-μs first transition, and totaling 0.49 fraction, is included in In2. The K + L1 + L2 mixture of the μs second transition, also totaling 0.49 fraction, is included in In3. The individual fraction values of L1 and L2 can only be obtained by fitting kinetic schemes to the b-spectra, as discussed below. The K + L1 + L2 mixture converts irreversibly into an intermediate, which also has an L-like spectrum and is included in In4. This intermediate state is called the N1 state. It is no longer in equilibrium with the K state and therefore it must be different from the L1 an L2 states by conformation and/or structure. The K, L1, L2, and N1 states together with 0.49 of the recovered R state, labeled R(2) in Fig. 1 *D*, form the backbone of the nonconductive cycle. As mentioned above, the N2, M, and R states in In5 cannot be assigned unequivocally to Cycle1 or Cycle2, they are the shared states. Separation of the R state formed in the early ms transition, R(In5), from the total recovered R makes our discussion easier to follow.

The above procedure of partitioning the spectral forms between two cycles in the WT protein follows directly from the assignment of the open channel state to the K-like spectral form. The alignment of the current trace with the time evolution of the K-like spectral form defines the parallel cycles fully and uniquely. The current alignments for the different proteins show that the K-like spectral form dedicated to the conductive cycle is equal to its amount contained in In4 and In5. Thus, the compositions of In4 and In5 are used to design the conductive cycle accurately. Replacement of the spectral forms present in the sequential intermediate spectra with intermediates, resulted in the two-cycle kinetic model. The other protein samples in this study also yield this type of two-cycle model, except the S97E mutant at low pH which will be dealt with separately. The results of the procedure are generalized in Table 1 where the letters *a*, *b*, *c*, and *d* refer to the fraction numbers of the K-like, L-like, M, and R spectral forms, respectively. In the analysis below, these letters are replaced by the actual numbers found in the composition matrices for the different proteins (11).

**Table 1:** Assigning spectral forms to intermediates of Cycle1 and Cycle2

	In1	In2	In3	In4	In5	In6
K-like	1	a2	a3	a4	a5	–
L-like	–	b2	b3	b4	b5	–
M	–	–	c3	c4	c5	–
R	–	–	–	–	d5	1

**Cycle1**						

K	a4	a4	a4	–	–	–
O	–	–	–	a4	a5	–
R	–	–	–	–	–	a4

**Cycle2**						

K	1-a4	a2-a4	a3-a4	–	–	–
L1 + L2	–	b2	b3	–	–	–
N1	–	–	–	b4	–	–
M	–	–	–	c4	–	–
R	–	–	–	–	–	a4

**Shared**						

N2	-	–	–	–	b5	–
M	-	–	–	–	c5	–
R	-	–	–	–	d5	–

### Kinetic interpretations of the conductive cycle

In the single chain models published earlier, the blue-absorbing L intermediate, identified with the open channel state, was presumed to be in equilibrium with the nonconductive M state (9,10). We show below that the two-step closure of the channel in the conductive cycle can also be presented as having a reversible step between the conductive red-absorbing O state and the nonconductive N2 or M state. However, the physical reality of such equilibria will be contested and the kinetics will be interpreted using the more realistic branching scheme model.

#### The contested equilibrium scheme

The conductive cycle outlined above starts with the K state converting irreversibly into the open channel state O, step K→O. The O state is present in both In4 and In5, thus it decays in two steps. Except, at low pH, the O state can convert into the N2, M, and R states, or any combination of them in the early ms fast decay step, thus the two-step O decay can be accomplished in numerous ways. Fortunately, at low pH the deprotonation of the Schiff base is not observed, and Cycle1 has only the K, O, N2, and R states interconnected. This greatly simplifies the kinetic problem because for four intermediates the scheme, including its reaction rates, can be successfully calculated by the algebraic method (15). There is no need to make guesses about the scheme.

**Figure 2:**
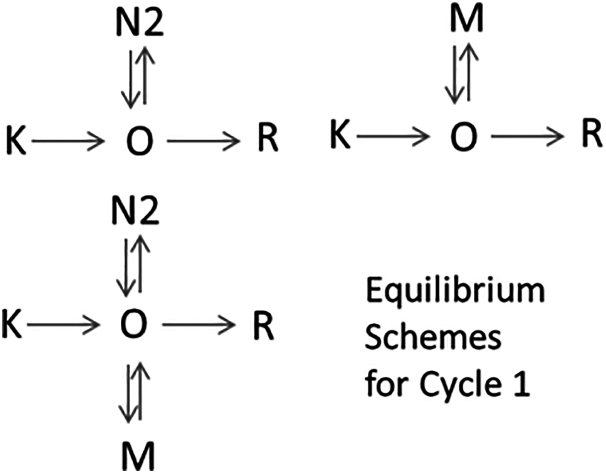
Cycle1 equilibrium schemes.

Making use of this advantage, we calculated the kinetic scheme for the WT protein at pH 5.5. The matrix calculation yielded a kinetic scheme that contains an irreversible K→O step followed by a branch. In the branch an irreversible O→R step is responsible for the fast O decay and an O↔N2 side equilibrium step for the slow O decay. The scheme shows that any two-step decay can be explained by a branching reaction step, in which a direct decay step constitutes one branch, while a side equilibrium step makes up the second branch. Because the shared states present in In5 can be combined in countless numbers of ways, there are countless numbers of schemes of this type, each with different sets of reaction rate constants for the branching step. This scheme pattern is shown in equilibrium schemes for Cycle1 (Fig. 2). At higher pH, the decay of the O state produces not only N2 but also M, and when both N2 and M are chosen as products the scheme becomes far more complex and may have two side equilibria. Scheme patterns of this type, three of which are shown in equilibrium schemes for Cycle1 (Fig. 2), were tested and found to describe the kinetic data perfectly.

Despite their attractive features, the equilibrium schemes can be contested. Although valid in the chemical sense, we argue that *neither of the equilibrium schemes* should be considered as the *acceptable mechanism of channel kinetics* for the following reasons. In the conductive Cycle1, the N2 and M states produced by channel closure are nonconductive. Their equilibria with the conductive O state would mean frequent switching between open and closed states of the channel in the same reaction chain. Considering the extensive structural rearrangements that accompany channel opening and closing, this picture is physically highly unrealistic. Once the channel is closed in the chain of events, it makes no sense to reopen it and close it again within the short time period of the reaction sequence, and do it repeatedly, as required by the concept of equilibrium. Moreover, if open and closed states of the channel could exist in equilibrium they would be at similar energy levels and, consequently, spontaneous opening of the channel in the dark would be a possibility. That clearly refutes the purpose of a light-sensitive channel. Because of these problems, the equilibrium schemes are not pursued and presented in more detail. However, we do not exclude equilibria between different protein conformers that produce open and closed states in their photokinetics.

#### The branching scheme

Rejecting the idea of equilibrium between the open and closed channel states, the reversible O↔N2 and O↔M reaction steps, which produced the mixture of the conductive O and nonconductive N2 and M states in the discussion above, should be reconsidered. The nonconductive states must have formed in an alternative way, called the irreversible branching mechanism. In it, the fast and slow O decay is no longer accomplished by a single molecule but by two different O molecules representing two conductive states, named O1 and O2. This conclusion applies to all proteins and conditions discussed in this work. We propose that *two isospectral but kinetically different states, O1 and O2, are present in the conductive photocycle and are responsible for the fast and slow current decays*. The O1 and O2 states may belong to a single conductive cycle or may have their own conductive cycles leading to the two-cycle and the three-cycle kinetic models, respectively.

#### The single conductive cycle: Cycle1

**Figure 3:**
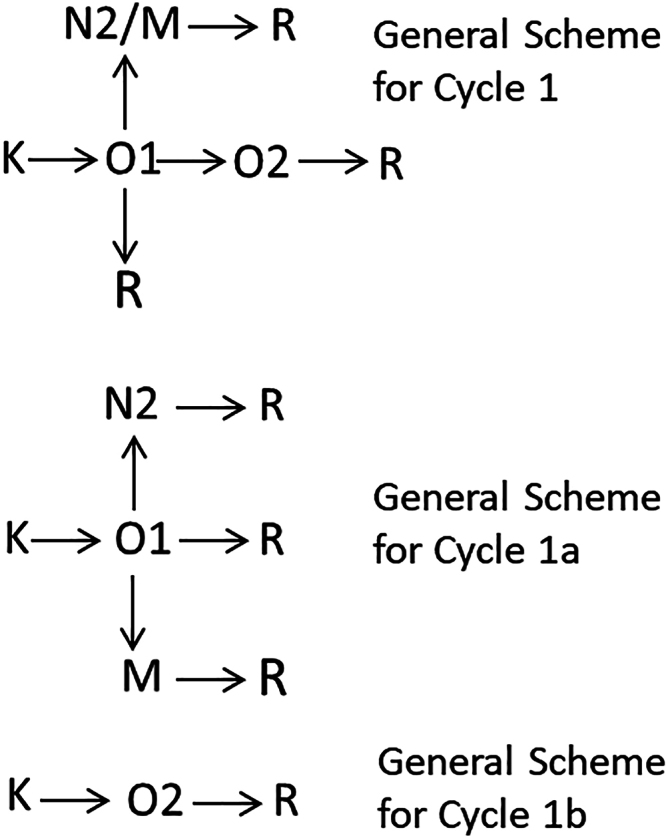
Cycle1 branching schemes.

The branching general scheme of Cycle1 in Fig. 3 starts with the K state converting irreversibly into the conductive O1 state. The conductive O1 state decays in an irreversible branching step into the conductive O2, the nonconductive N2 and M states, and the final R state. The N2 and M states decay into the R state in separate branches. The branching step of the fast-decaying O1 state, which corresponds to the fast current decay, has an overall rate equal to the fourth experimental apparent rate (the early ms lifetime). Because the overall rate of the branching step, k_app_(4), is the sum of the rates of the individual branches, the latter can be calculated based on the relative amounts of the intermediate states formed in the branch: k_i_ = k_app_(4) × fr_i_/Σ fr_i_. The O2→R, N2→R, and M→R branches have the same rates equal to the fifth experimental apparent rate (late ms lifetime). This corresponds to the slow current decay. All reaction rates in the schemes derived for Cycle1 thus can be obtained for any desired combination of the shared states.

#### The two conductive cycles: Cycle1a and Cycle1b

Because the O1 and O2 states decay differently, they are assumed to be physically different. They may belong to separate cycles, thus splitting Cycle1 into two, Cycle1a and Cycle1b. Compared with Cycle1, the amplitudes of the intermediate states in Cycle1a get reduced by the amount of the O2 state allocated to Cycle1b. In this interpretation, as seen in the general scheme for Cycle1a in Fig. 3, the O1 state in Cycle1a no longer converts into the O2 state in the early ms transition. It decays only into N2, M, and R shared nonconductive states. Both the N2 and M states decay into the R state with the late ms lifetime as in Cycle1. The first step in Cycle1b in Fig. 3 is the K→O2 step, which is assumed to have the same rate as the K→O1 step in Cycle1a. The O2 state decays in a single O2→R step with the late ms lifetime. Cycle1b does not share any intermediates with Cycle2 or Cycle1a, it is uniquely defined and its scheme is fairly simple: K→O2→R. The assignment of O1 and O2 to two different conductive cycles, and thus creating the three-cycle model, is based on physical reasons alone. Both the two-cycle and three-cycle models describe the experimental data accurately.

Is the two-cycle or the three-cycle model more realistic? In the Cycle1 scheme the open channel O1 state decays into nonconductive N2 and M states and into another, slower decaying conductive O2 state simultaneously. The first is accompanied by closing the channel, while the second leaves the channel open. This is not very realistic physically. Thus it is more likely that the reaction follows two conductive cycles. Also, analysis of the S97E mutant data at pH 5.6 discussed below supports the two conductive cycle mechanism. This, however, is not sufficient to reject the Cycle1 scheme entirely and it is included in the analysis below.

### Kinetic interpretations of the nonconductive cycle

**Figure 4:**
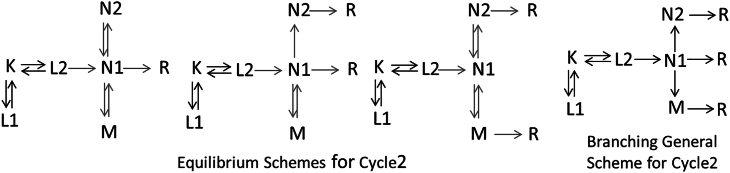
Cycle2 equilibrium and branching schemes.

The nonconductive cycle is not involved directly in channel opening and closing so its details seem to be of lesser importance for understanding the channel function. The backbone of the nonconductive cycle was already discussed above. It starts with a two-step equilibration between the K and the L1 and L2 states, followed by an irreversible step that produces the N1 state. The first part of the mechanism thus can be presented in the following way: L1↔K↔L2→N1. As in Cycle1, there are two more steps to complete Cycle2. In the faster of the two steps, N1 branches into the shared N2, M, and R states with the fourth, early ms, experimental apparent lifetime. The complete recovery to R occurs with the fifth, late ms, experimental apparent lifetime. N1 can be connected to the shared N2 and M states reversibly or irreversibly, yielding the schemes shown in Fig. 4 as equilibrium schemes for Cycle2 and branching general scheme for Cycle2, respectively. Albeit, there are no restrictions that would prevent accepting reversible late reaction steps in the nonconductive cycle, in our further discussion we give preference to the irreversible branching pattern, which is also followed by Cycle1 and Cycle1a. The conductive and nonconductive cycles share the last two apparent rates, which makes it plausible that they also share the branching decay pattern.

The Cycle2 nonconductive schemes for the different proteins are more complex than the conductive cycle schemes. Some of the reaction rates in them are best obtained by using the scheme-fitting method. The fit reproduces the experimental apparent rates and b-spectra through the eigenvalues and eigenvectors of the kinetic matrix, respectively, using the spectral forms for the spectra of states in the fit. The apparent rates fitted are those found in the global exponential fit (11). Because the schemes for Cycle1 and Cycle2 complement each other, the b-spectra to be fitted for Cycle2, ***bsf***, are calculated from the experimental b-spectra (11), ***bs***, by subtracting the b-spectra corresponding to the Cycle1 scheme: ***bsf = bs***–***E***×***W***_***cy1***_. Here, ***E*** is the matrix of spectral forms serving as spectra of the states, and ***W***_***cy1***_ is the eigenvector matrix of the kinetic matrix for Cycle1. The rate constants of the steps branching from N1 in the branching scheme can be calculated the way described above for the O branching in Cycle1.

### Kinetic schemes with reaction rate constants for the WT and mutant proteins

**Figure 5:**
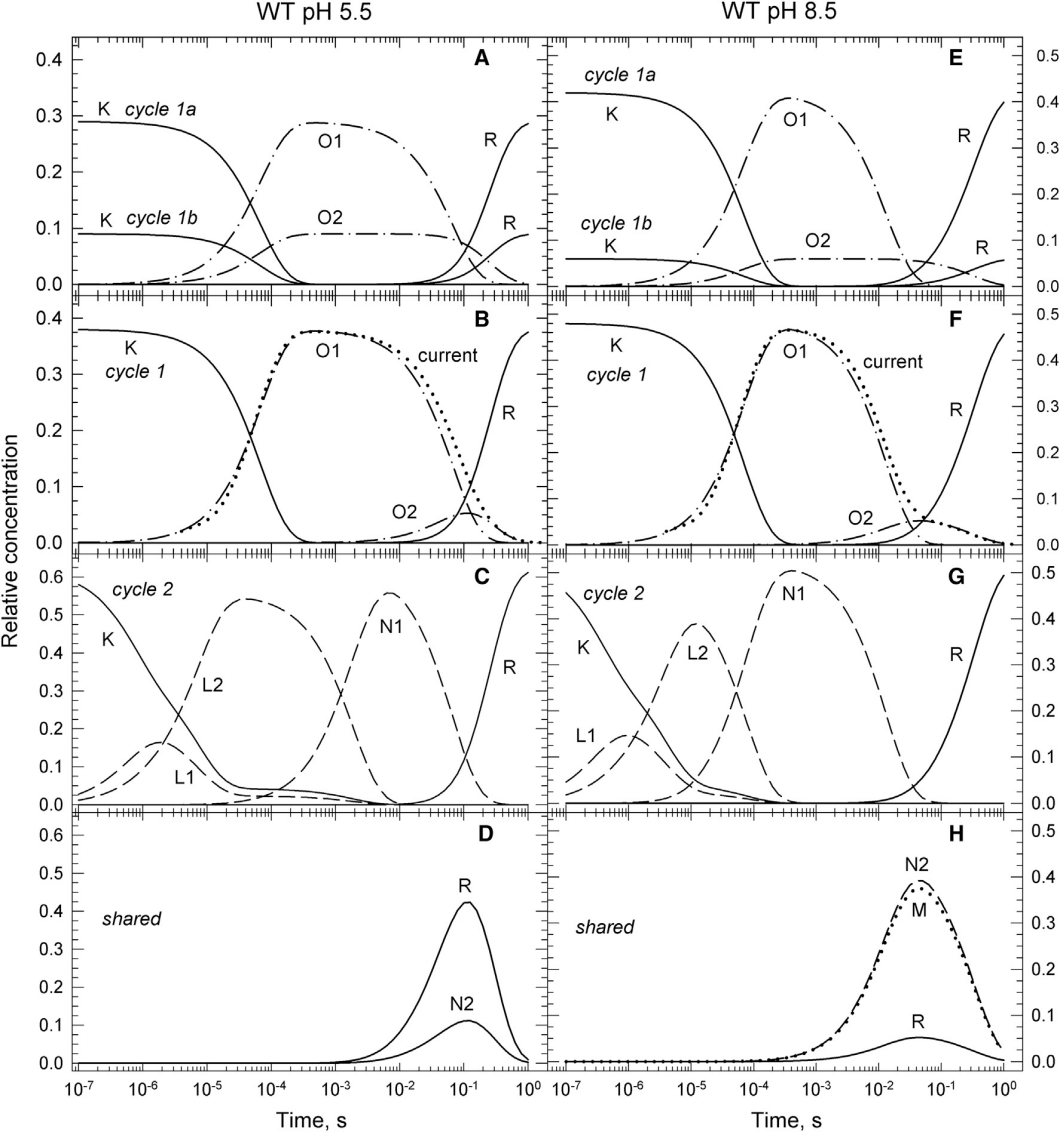
Time evolution of intermediate states of the conductive and nonconductive parallel photocycles for the WT protein at pH 5.5 (*A*–*D*) and 8.5 (*E*–*H*) calculated based on the reaction rate constant shown in the corresponding schemes. (*A* and *E*) The time dependence of the K, O1, and R states that belong to Cycle1a and that of the K, O2, and R states that belong to Cycle1b. (*B* and *F*) The concentration profiles of the intermediate states of Cycle1. The current trace (*dotted line*) follows the sum of O1 and O2. The O1 and O2 are two consecutive conductive states. (*C* and *G*) The concentration profiles of the intermediate states of Cycle2. (*D* and *H*) The states shared between the nonconductive Cycle2 and the conductive Cycle1 or Cycle1a. There is no M state at pH 5.5.

The kinetic schemes discussed above in general terms were applied to all of the proteins in this study, and found to describe the experimental data quantitatively. The results are presented below. Kinetic schemes are best understood by viewing the time-dependent concentrations of their intermediates. Because Cycle1b is uniquely defined, it is the easiest to present. Both Cycle1a and Cycle1 share intermediate states with Cycle2 in an undefined way, which poses problems to their presentation. To overcome this, we show the time evolutions of the states that are not shared, and thus uniquely defined for the cycles, separately from those of the shared states. The shared states include not only the N2 and M states, but also that part of the R state that forms with the early ms lifetime and contribute to In5. For illustration purposes, and only for that, the R state is split into early and late components. In the figures displaying the intermediate concentrations, Figs. 5, 7, 9 and 11, Cycle1 and Cycle2 of the two-cycle kinetic model are presented in *B* and *C*, or *F* and *G*, respectively. The Cycle1a and Cycle1b of the three-cycle kinetic model are shown in *A* or *E*, and the shared states in *D* or *H*.

#### WT at pH 5.5 and 8.5

Because the currents at these pH values were not published, the fractions of the K-like spectral form assigned to the conductive Cycle1 are based on the compositions of In4 and In5, as mentioned above. At pH 5.5, In4 and In5 contain 0.38 and 0.09 K-like forms, respectively. Of this, 0.29 is O1 and belongs to Cycle1a, and 0.09 is O2 and belongs to Cycle1b. At pH 8.5, the total conductive K-like fraction is 0.48, of it 0.42 is O1 and belongs to Cycle1a and 0.06 is O2 and belongs to Cycle1b. The time evolutions of the K, O2, and R states of Cycle1b, and the K, O1, and R states assigned exclusively to Cycle1a, are shown in Fig. 5, *A* and *E* for the WT at pH 5.5 and 8.5, respectively. Fig. 5, *B* and *F* display the time evolutions of states in Cycle1, and Fig. 5, *C* and *G* in Cycle2. The shared states are in Fig. 5, *D* and *H*. The current traces, dotted lines, shown in Fig. 5, *B* and *F* serve to illustrate the connection between the current and the intermediate state kinetics. The traces shown are derived from the average current trace recorded at pH 7.4. At pH 5.5, the fast decay of the current was slowed down by a factor of 1.8 and its amplitude was reduced by 10%, while accelerating the slow decaying component by a factor of 1.2. The trace at pH 8.5 was created by accelerating the fast decay rate by a factor of 3.3, in accord with the published information (6). The small sized slow component was further reduced by a factor of 2.2.

**Figure 6:**
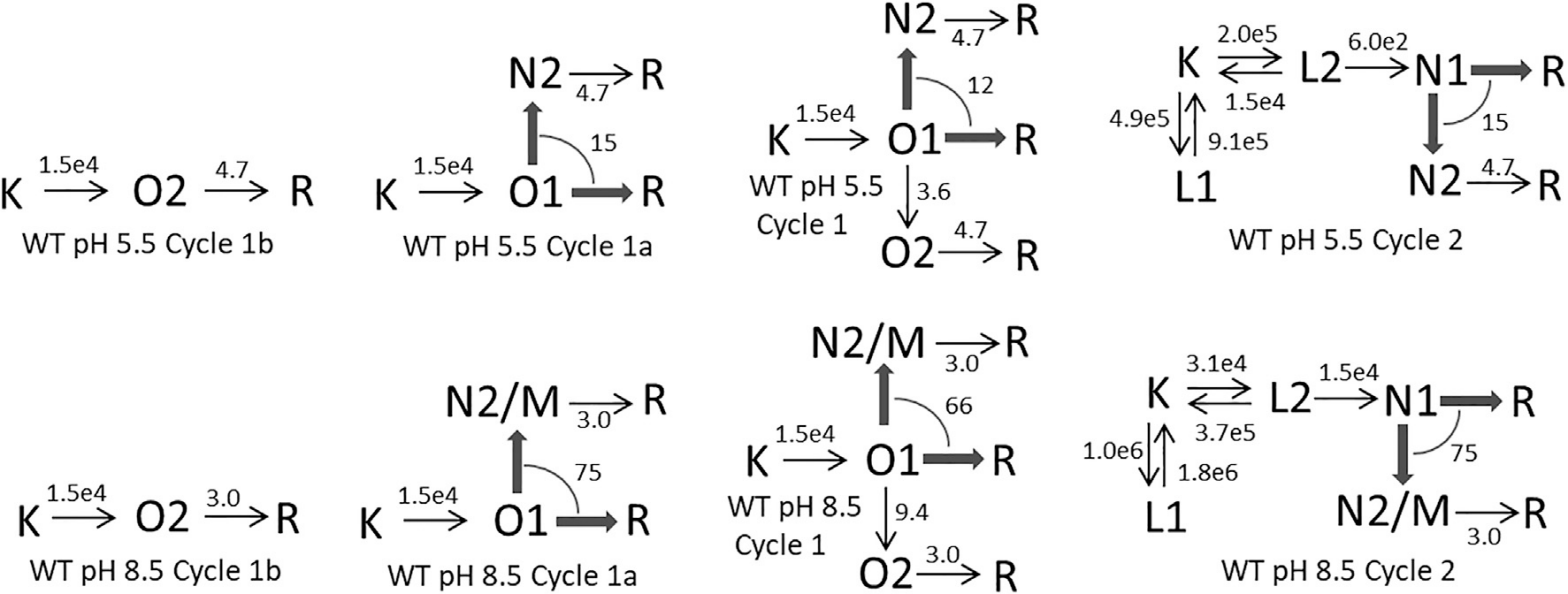
WT pH 5.5 and 8.5 kinetic schemes.

The kinetic schemes shown in Fig. 6 for the cycles at pH 5.5 and 8.5 contain the reaction rate constants (s^−1^) obtained by scheme fitting and direct calculation as discussed above. The thick arrows are the potential branching steps of the O1 and N1 decays and have the overall rates indicated on the arcs. The rates of the individual branching steps can be calculated as described above. To get the full picture of the time evolutions for a particular choice of sharing, the time evolutions of Cycle1a, Cycle1, and Cycle2 shown for pH 5.5 and 8.5 should be complemented by adding to them the appropriate portions of the concentration profiles of the shared states in Fig. 5, *D* and *H*, respectively. In the full picture the artificially separated parts of the R state have to be combined.

The schemes for Cycle1 and Cycle2 at pH 5.5 are simpler than the general schemes, the M→R branches in the general schemes are missing. Also, if all 0.19 fraction of N2 is assigned to Cycle2, both Cycle1 and Cycle1a miss the N2→R recovery branch of the general scheme and become uniquely defined. At pH 8.5 the schemes presented are as complex as the general schemes are.

#### WT at pH 7.4

**Figure 7:**
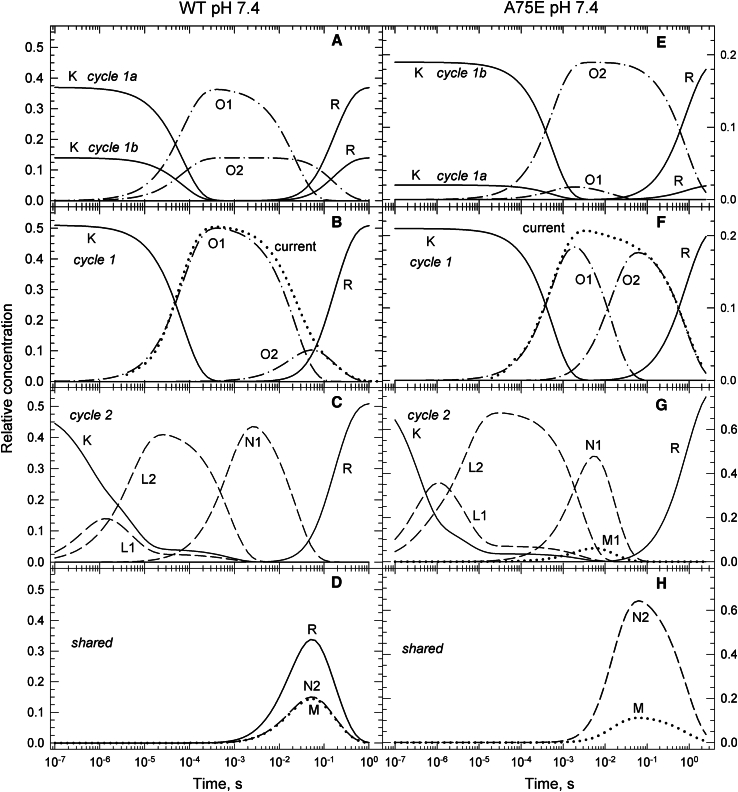
Time evolution of intermediate states of the conductive and nonconductive parallel photocycles for the WT protein at pH 7.4 (*A*–*D*) and for the A75E mutant at pH 7.4 (*E*–*H*) calculated based on the reaction rate constant shown in the corresponding schemes. The concentration profiles presented are analogous to the ones described in Fig. 5. The only exception is the presence of an early M state in the A75E kinetics assigned to Cycle2 and shown in (*G*) by the dotted line.

The time evolutions of intermediate states assigned exclusively to Cycle1 and Cycle2 are displayed in Fig. 7, *B* and *C*, respectively, and the shared states are shown in Fig. 7 *D*. The current displayed in Fig. 7 *B* (*dotted line*), which follows the sum of the conductive O1 and O2 intermediate concentrations almost perfectly, is a modified version of the average current measured under positive and negative potentials. In it the fast and slow decay components were amplitude corrected by factors of 0.9 and 1.1, and accelerated by factors of 1.7 and 1.6, respectively. Differences of this magnitude between electrophysiological and optical data are fairly common.

**Figure 8:**
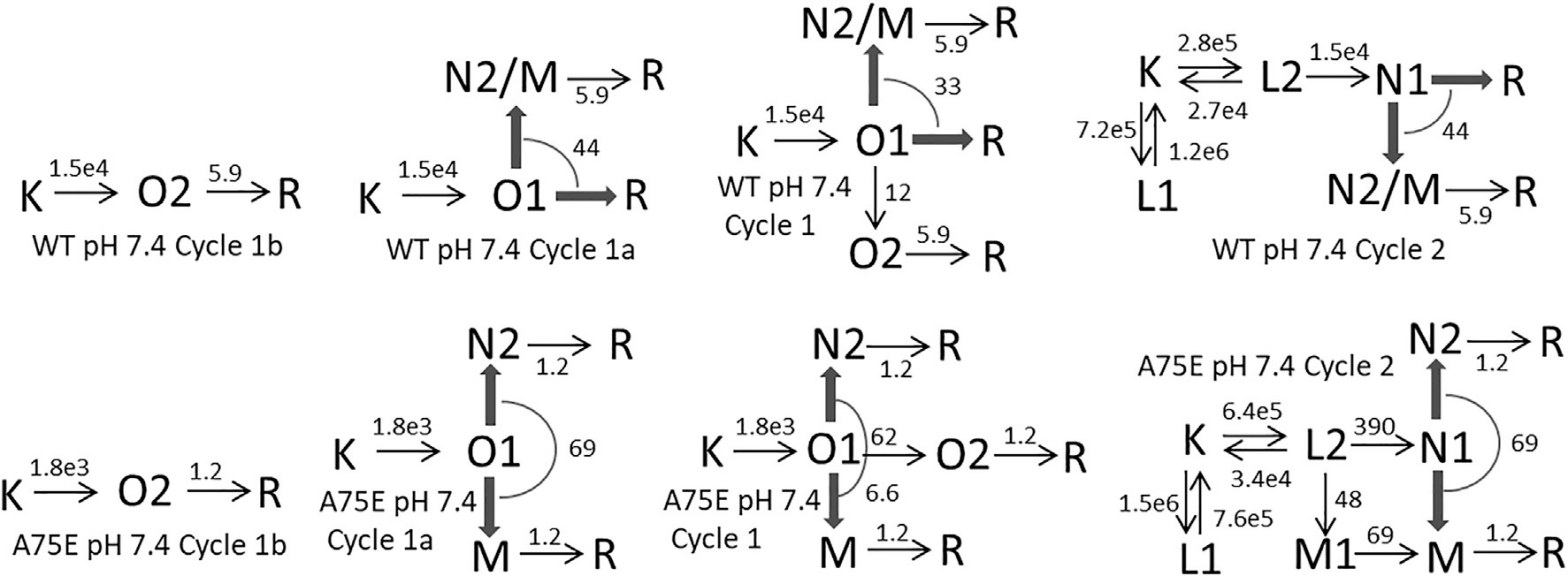
WT pH 7.4 and A75E pH 7.4 kinetic schemes.

As above, the kinetic schemes shown in Fig. 8 for Cycle1a, Cycle1b, Cycle1, and Cycle2 contain the rate constants. The thick arrows point toward potential side branches of recovery and have the overall rate displayed on the arc. In Cycle1a 0.37 O1 converts into the shared N2, M, and R states. Most of the N2 + M + R = 0.37 combinations yield the Cycle1a general scheme. A few, however, may simplify the scheme by removing the M→R branch, the N2→R branch, and the O1→R fast R recovery step. Similarly, some of the Cycle2 schemes may lack the N2→R and M→R branches.

The kinetics at pH 5.5, 7.4, and 8.5 are not dramatically different. Deprotonation of the Schiff base (M formation) at pH 7.4 and 8.5 accelerates the O1 decay and reduces the amount of R recovered in the fast step by redirecting the recovery to the slower M→R and N2→R side branches.

#### A75E at pH7.4

In this mutant alanine 75, located at a distance of around 0.9 nm from the chromophore and close to the extracellular surface, is replaced by glutamic acid. The replacement affects both the channel current and the photocycle kinetics (Fig. 7, *E*–*H*). The photocurrent shown in Fig. 7 *F* (*dotted line*) was recorded at −30 mV and has much bigger slow decay component than seen for the WT. The O1 and O2 concentrations in Cycle1 (Fig. 7 *F*) follow the published current almost perfectly. The amplitude of the O2 state in Cycle1b is much higher than that of the O1 state in Cycle1a (Fig. 7 *E*), which is opposite to the trend seen in the WT protein. No R state is recovered from O1 in Cycle1 and Cycle1a, and from N1 in Cycle2. Interestingly, both In4 and In5 contain the M state, an early M1 and a late M, respectively. In Cycle1 (Fig. 7 *F*), a big portion of the O1 state converts to O2, and only a very small fraction of it into M and N2. Most of the shared M2 and N2 states in Fig. 7 *H* thus belong to Cycle2, to which the early M1 is also assigned (Fig. 7 *G*). Cycle2 in Fig. 8, having the early M1 state included in it, is more complex than the general scheme. It shows simultaneous formation of the M1 and N1 states in the early branching step from L2, followed by M1→M→R and N1→N2→R decay paths. The late M state is also formed from N1 as in the general scheme. Both Cycle1a and Cycle1 in Fig. 8 follow the general scheme lacking the O1→R early recovery step. Most of the R state recovers in the slow O2→R step.

### Kinetics of the low-conductance mutant proteins

The WT protein and the A75E mutant have normal conductance and display similar levels of the conductive and nonconductive cycles. For the low-conductance D234N and S97E mutants, which played pivotal roles in choosing the intermediate responsible for the open channel state, this is not expected. Their kinetics is discussed below.

#### D234N at pH 7.4 and 4.5

**Figure 9:**
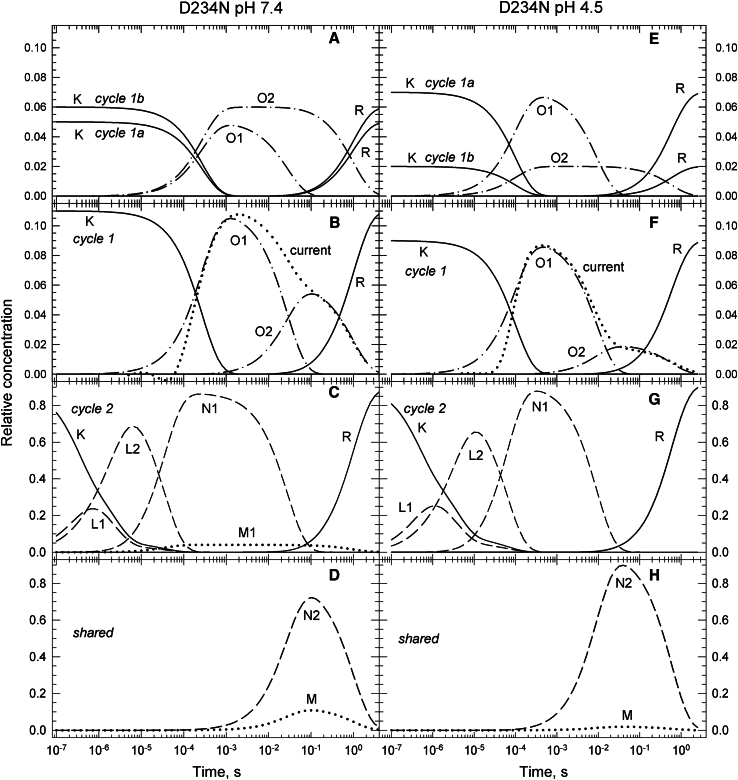
Time evolution of intermediate states of the conductive and nonconductive parallel photocycles for the low conductance D234N mutant at pH 7.4 (*A*–*D*) and 4.5 (*E*–*H*) calculated based on the reaction rate constant shown in the corresponding schemes. (*A* and *E*) Concentration profiles of the states in Cycle1a and Cycle1b. (*B* and *F*) Same for Cycle1. (*C* and *G*) Intermediate states of the nonconductive Cycle2. Note the presence of the early M state in (*C*). (*D* and *H*) Time dependence of the shared states. Note the absence of the R state, D234N recovers in one slow step.

The D234N is a low-conducting variant displaying well-separated fast- and slow-decaying current components (9). To get a good match to the O1 and O2 intermediate state concentrations at pH 7.4 (Fig. 9 *B*), both the fast and slow components of the current were decelerated by factors of 3.7 and 1.9, respectively, while keeping their amplitude ratio unchanged. The current at pH 4.5 is quite different from that at pH 7.4 in both the decay rates and the amplitudes of its fast and slow components. The concentration profiles of the O1 and O2 intermediate states (Fig. 9 *F*) follow this trend and match the current trace almost perfectly.

**Figure 10:**
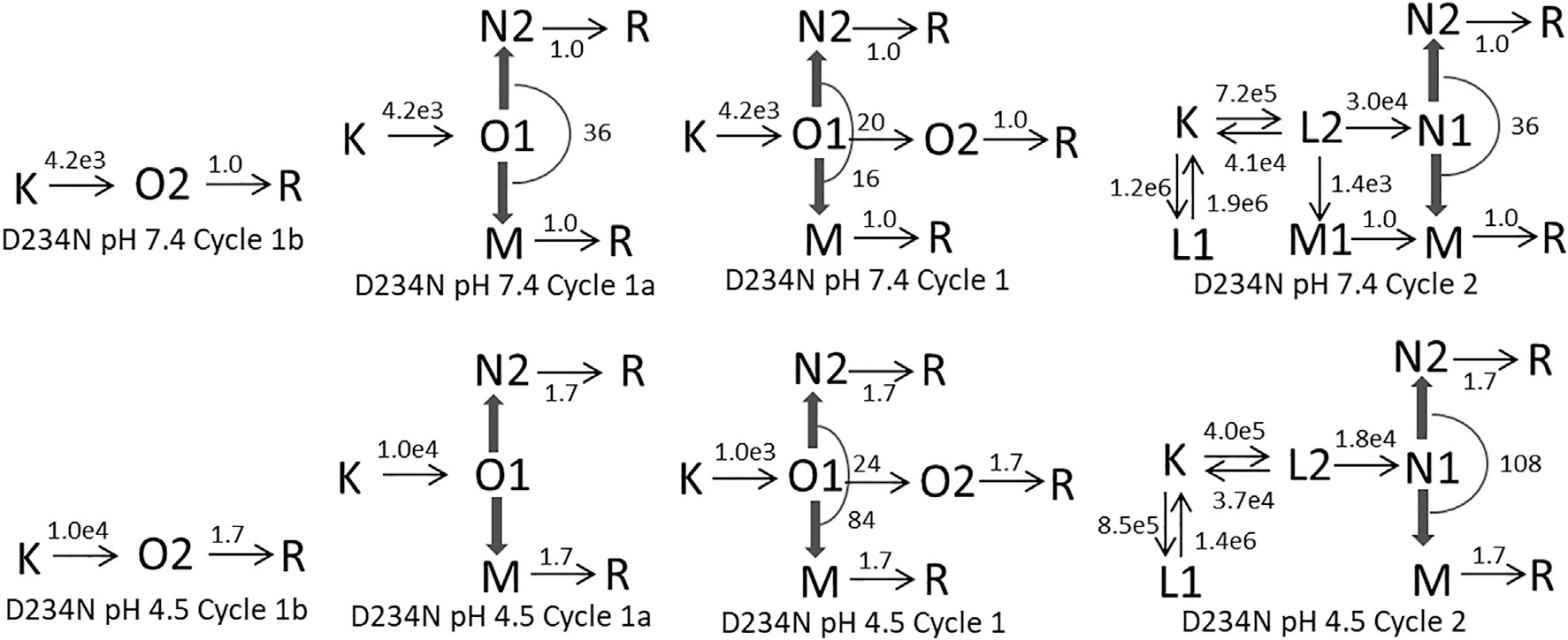
D234N pH 7.4 and 4.5 kinetic schemes.

Similarly to A75E, the D234N mutant at pH 7.4 shows an early M1 state in In4 and a late M state in In5. Also, the R state is recovered only in the last reaction step. The charge movement during the early deprotonation of the chromophore induces a current transient, typically observed in the BR and cation channelrhodopsin photocycles, which distorts the rising part of the channel current (Fig. 9 *B*). Cycle1 starts with 0.11 fraction of the K state. It converts to the O1 state, of which a fraction of 0.05 is lost in the fast decay step to either of the nonconductive N2 and M states, or to their combination. This 0.05 fraction of the O1 state constitutes the O1 state of Cycle1a, while the remaining 0.06 fraction is the O2 state of Cycle1b. Similarly to A75E, the O2 state has somewhat higher amplitude than the O1 state. Since the conductive fraction is small, most of the shared N2 and M content of In5 belong to Cycle2, together with the early M1 state which is of small amplitude. The kinetic schemes for Cycle1 and Cycle2, shown in Fig. 10 with the reaction rates, are similar to the general schemes without the O1→R and N1→R branches, but having an extra M1-branch from L2 in Cycle2. The latter is arranged in the same way as the M1-branch in Cycle2 for the A75E mutant.

At pH 4.5, the conductive Cycle1 starts with 0.09 of the K state which converts to the O1 state. The shared states in In5 are dominated by the 0.96 fraction of the N2 state. There are also 0.02 fractions of both the O2 and the M states, of which the amount of M is on the borderline of detection in that wavelength range. Since the M state is present at pH 7.4, and most of it belongs to Cycle2, a trace amount of it may as well form at low pH. The schemes for Cycle1 and Cycle2 are similar to the ones at pH 7.4 and they also miss the O1→R and N1→R fast decay steps. The O1 state in Cycle1a has a fraction value of 0.07, which is much higher than the 0.02 fraction of the O2 state in Cycle1b. This is similar to the trend seen in the WT protein.

#### S97E at pH 7.4

**Figure 11:**
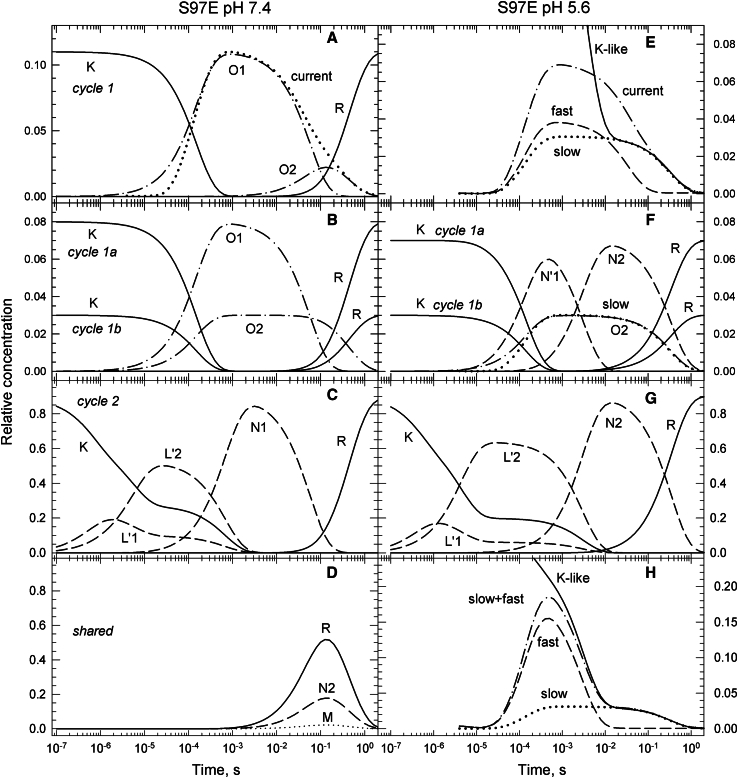
(*A*–*D*) Time evolution of intermediate states of the conductive and nonconductive parallel photocycles for the low conductance S97E mutant at pH 7.4 calculated based on the reaction rate constant shown in the corresponding schemes. (*A*) The conductive Cycle1 states at pH 7.4. (*B*) Intermediate states of the conductive Cycle1a and Cycle1b at pH 7.4. (*C*) Same for Cycle2 at pH 7.4. Note the early L′ isospectral states have different, less blue-shifted absorption spectra from the N1 state. (*D*) Shared states at pH 7.4. (*E*–*H*) S97E mutant unusual kinetics at pH 5.6. (*E*) The time evolution of the fast component of the channel current recorded at pH 7.4 (9) does not follow the K-like spectral form at pH 5.6. The O1 conductive state is missing at pH 5.6. (*F*) The O1 state is replaced by the N′1 state having L′-like spectrum, considered to represent a nonconductive state. Time evolution of the K, N′1, N2, and R states in the unusual nonconductive Cycle1a, and the K, O2, and R states of the usual conductive Cycle1b. (*G*) The nonconductive Cycle2 with the K, L′1, L′2, N2, and R states. Note there are no shared states at pH 5.6. (*H*) Forced alignment between the spectral and current traces requires changing the amplitude and decay rate of the current’s fast component by factors of 5 and 12, respectively, which is unrealistic and unacceptable.

**Figure 12:**
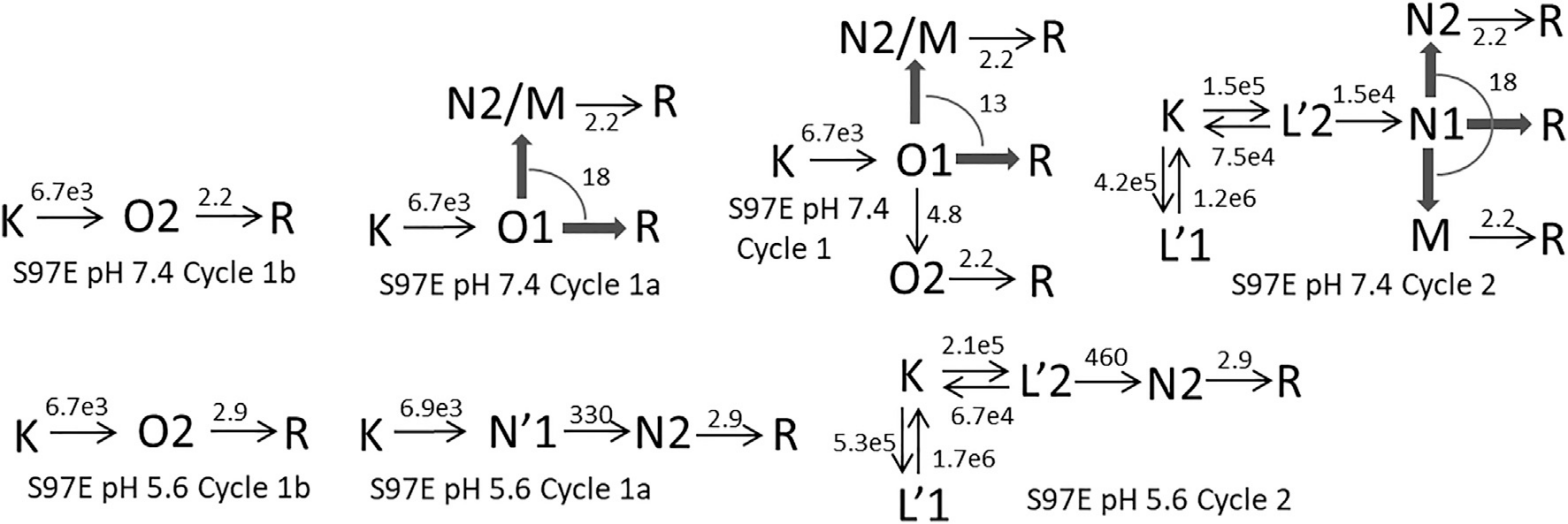
S97E pH 7.4 and 5.6 kinetic schemes.

This mutant shows even less conductance than the D234N mutant does. The spectrum of the early L′1 and L′2 states is almost identical to the spectrum of the recovered state, which is only very slightly blue shifted from the dark spectrum. This makes S97E different from the other proteins discussed. The usual and more blue-shifted L-like spectrum appears at later times. The N1 and N2 states in the photocycles have the normal L-like spectra. Despite the presence of a carboxyl group, a potential proton acceptor near the protonated Schiff base, it forms little M state, less than the WT. Its late kinetics is quite different from that of the low-conducting D234N mutant; it shows large amounts of R recovered in the early ms step. A fraction of 0.11 of the K state belongs to the conductive Cycle1 and converts into the O1 state (Fig. 11 *A*). In the fast recovery step, a fraction of 0.08 of the O1 state decays into the shared N2, M, and R states, and a fraction of 0.03 into the O2 state. The concentration profiles of the O1 and O2 states are well matched to the current trace (9) (Fig 11 *A*). Cycle1a has 0.08 O1 and Cycle1b has 0.03 O2. The time evolutions of the intermediate states in Cycle1a and Cycle1b are shown in Fig. 11 *B*. Because of the small amplitude of the conductive cycle, most of the shared N2, M, and R states in Fig. 11 *D* belong to the nonconductive Cycle2 in Fig. 11 *C*. The kinetic schemes shown in Fig. 12 for Cycle1, Cycle1a, and Cycle2 have the patterns of the general schemes.

#### S97E at pH 5.6

##### Support for two independent conductive cycles

The current profile is not available, and the trace recorded at pH 7.4 (9) is hard to modify in a way to follow the concentration profile of the K-like spectral form (Fig. 11 *E*). The fast and slow components of the current are usually different by a factor of around 10. Here, the two slowest decays of the K-like spectral form, apparent lifetimes 3.0 and 350 ms, differ by a factor of 110. While the slow-decaying current component, dotted line, of S97E, recorded at pH 7.4, can be aligned to the spectral trace, solid line, almost perfectly (Fig. 11, *E* and *H*), the fast decaying component of the current, dashed line, must be accelerated by a factor of at least 12 to approach the decaying part of the K-like spectral form, as shown in Fig. 11 *H*. Note that shifting the fast current component without increasing its amplitude by a factor of 5 would not solve the problem and would cause unusual complications in Cycle2 early kinetics, including the early equilibration processes between K and L′. Thus, accelerating the fast current component does not seem to yield a realistic solution.

It is far more realistic to accept that the O1 intermediate state is missing at low pH and the composition of In4 has to be reexamined. We propose that the 0.30 K in In3 is shared between three cycles and undergoes the following transitions: 0.07 of it belongs to Cycle1a and converts irreversibly into the nonconductive N′1 state with L′-like spectrum, 0.03 of it belongs to Cycle1b and converts into the conductive O2 state, and 0.20 K belongs to Cycle2 and stays silent in the transition between In3 and In4. Thus, In4 contains 0.07 of the N′1 state belonging to Cycle1a, 0.03 of the O2 state belonging to Cycle1b, and 0.20K + 0.70(L′1 + L′2) belonging to Cycle2. The difference between the photocycles at pH 5.6 and 7.4 is that in In4 the 0.07 nonconductive N′1 state replaces the usual conductive O1 state. In the transition between In4 and In5 the K + L′1 + L′2 intermediate state mixture of Cycle2 and the N′1 state of Cycle1a convert into two N2 states, which are isospectral and have the usual L-like spectra. The processes mentioned are going simultaneously in three parallel cycles in Fig. 12. Because there are no M and R states in In5, the reaction rates in the three cycles can be assigned unambiguously. The concentration profiles of the intermediate states for Cycles 1a, Cycle1b, and Cycle2 are shown in Fig. 11, *F* and *G*, respectively. This interpretation of the kinetics is consistent with the three-cycle model.

One interesting feature of the S97E kinetics at pH 5.6 is the closed channel state in Cycle1a. The K→O transitions in the other proteins, which are usually assigned to the channel opening, are spectrally silent; leaving no easily detectable spectral traces behind. The lifetimes of these silent transitions are borrowed from current measurements. Here, the analogous K→N′1 transition of Cycle1a is not silent spectrally, it is easily detectable. We found 146 *μ*s for its lifetime. Fitting the current recorded at pH 7.4 with four exponentials gave 132 *μ*s lifetime for its increase. The agreement between the lifetimes supports the interpretation of the unusual kinetics observed at low pH for the S97E mutant.

Even though the contribution by the conductive cycle to the kinetics of the S97E mutant at pH 5.6 is relatively small and should be taken with caution, the interpretation given above raises a fascinating question: what is missing at pH 5.6 that was essential to open the channel at pH 7.4 in Cycle1a?

### The role of the M intermediate in anionchannel kinetics

The lack of deprotonation at low pH clearly shows that *M is not a necessary component of the channel closing kinetics.* Proton transfer from the Schiff base to the acceptor is a crucial event in BR function and happens at the early stages of the kinetics, right after the L intermediate. In anion channelrhodopsin such an event is undesirable because it would reduce the positive charge density at or near the channel. Indeed, early M formation is completely absent in the WT protein and happens only to a small extent in some of the “less perfect” mutants. However, during recovery, when the channel has already been closed, proton acceptors may approach the Schiff base temporally. The concentration of these temporary proton acceptors is higher at elevated pHs; thus, protein conformations with both protonated (N2) and unprotonated Schiff bases (M) coexist. The only common feature shared between the late M state of the anionchannel and the M of BR is that the Schiff base is unprotonated in both of them.

### The shape of the current signal and the rectifying A75E mutant

While reaction kinetics is believed to be tightly connected to the opening and closing of ion channels, it does not solely control the channel current amplitude and time-dependent profile; other factors are also important (16). The current in an open channel depends on the ion concentration at the entrance (mouth) of the channel, on the electrochemical mobility of the ion, and on the strength of the electric field that drives the ion across the membrane. Predicting the current amplitude is a very complex task because the ion concentrations and the electric field gradient are coupled quantities. In addition, the acting electric field is the superposition of the internal and external field components. It is not surprising that the current profiles under positive and negative potentials look somewhat different, or the zero current potential is different for the different proteins due to the asymmetrical charge distributions surrounding the channel. Because of the many factors that determine the current shape, fitting exponentials to the current trace and identifying each of the exponential components with a reaction step may lead to false results. Even a correct model of the reaction kinetics may not describe every detail of the experimental current profile.

The kinetic model does not explain the rectifying properties of the A75E mutant either. We believe that the property is connected to surface charges and not to reaction kinetics. Surface groups, such as carboxyl and amino groups, can have a significant effect on channel current by modifying the distribution of ions adjacent to the membrane surface at which they are located. The extra carboxyl group of A75E near the channel entrance may dissociate to some extent and its negative charge may repel the chloride ions and create a local surface layer depleted of Cl^−^ at the channel end where it is located. In this depleted layer the supply of Cl^−^ ions from the bulk to the channel entrance may become diffusion limited and no longer adequate to maintain a voltage-dependent channel current, i.e., increasing the applied potential is no longer accompanied by increase in the channel current. At the opposite end of the channel, the distant Cl^−^ ions are much less affected by the negative carboxyl group due to the longer distance, and also because the negative carboxyl group and its positive counterion act together as a dipole. The Cl^−^ current from the opposite side of the membrane is fairly normal. The voltage dependence of the channel current, thus, resembles that of a rectifying diode, as reported for this mutant (14). The charged group may also produce an electric field big enough to affect the nearby Schiff base. However, the effect is, most likely, independent of the direction of the applied potential and plays no significant role in the rectifying.

### The use of BR nomenclature and the differences between *Gt*ACR1 and BR photocycles

In naming the intermediates of the *Gt*ACR1 photocycles we followed the familiar BR nomenclature. Using the familiar letters does not mean that the intermediates of the *Gt*ACR1 photocycle are identical to those of the BR photocycle. The intermediates of *Gt*ACR1 serve different purposes than those of BR, and are not expected to have the same structures. Thus, naming an intermediate M, N, and O does not mean we assign to it the function and structure of M, N, and O in the BR photocycle; we use the names merely because they are well known and easy to associate with. The letter chosen refers to the position of the absorption spectrum relative to that of dark form, or to the time of emergence of the intermediate in the photocycle. We name the first nanosecond intermediate K and the ones forming on the *μ*s timescale L, as in BR kinetics. The intermediates present in the ms time window are named N and O, and are considered isospectral with, but structurally different from, L and K, respectively. They may or may not be preceded or followed by Schiff base deprotonation. In choosing a name, the absorption spectrum may have more preference than timing. An example of this is the M intermediate, which follows L and precedes N and O in the BR photocycle, but it is one of the last ones in the *Gt*ACR1 photocycle or is completely missing. In the BR photocycle, there is no direct analog to the recovered R intermediate in our model. We consider anion (*Gt*ACR1) and cation (*Ps*CCR2) channelrhodopsins to be members of the family of proteins that have somewhat different working and resting conformations. The events following the recovered R intermediate constitute the bridge between the two, rather than accommodating the N and O intermediates, as suggested (9,10).

The *Gt*ACR1 photocycle is significantly different from the photocycles proposed for BR, it has different steps and order of intermediates. While the mechanism of BR is still debated (17), our model of the *Gt*ACR1 kinetics gives a quantitative description of the events and reproduces the experimental data from which it was derived. It is also consistent with the electrophysiological results.

### Channel function and reaction cycles

The parallel cycles presume two (two-cycle model), or most likely three (three-cycle model) different protein conformations, each following its own reaction path. These conformations presumably exist before photoexcitation rather than being formed following relaxation from the excited state. Conformational differences may or may not be reported by the UV-vis spectra, which are sensitive only to the environment at or near the chromophore. While it is an essential part of the photochemistry, the retinal chromophore may not solely determine the structure of the protein, or the steps in the reaction path on which it is reporting. Other factors are likely to be involved.

The shared intermediate states present at the end of the photocycle prevent unambiguous assignment of reaction rates to all reaction steps. This should not create any confusion in the interpretation of the channel kinetics. Although spectrally different, the N2 and M states may well have the same conformation, be the same molecule with two faces, which differ only in the protonation state of the chromophore, as suggested by the pH-dependent population of the M state. Thus, the early ms decay step may be rather simple: the O1 and N1 states branch into R and this mixed N2/M state.

Interestingly, the normal and the low-conductance protein variants follow similar parallel cycles. It is the extent to which the conductive and nonconductive cycles contribute to the kinetics that puts them into different categories. The conductive and nonconductive cycles share the last two apparent rates, which may not be coincidental. Rather, it may suggest some kind of coupling or synchronization between the two in the ms time window. The ratio of the conductive and nonconductive cycles for the WT protein is near 1:1. The WT protein is likely to be optimally designed for its function, which raises the question whether the similarity of the kinetic patterns and the 1:1 ratio reflect the dimer structure of the protein, in which the two molecules work together, perhaps in different cycles, to accomplish the channel function.

## Conclusion

The current view on channelrhodopsin *Gt*ACR1 kinetics presumes a BR-like photocycle and associates the open channel state with a blue-shifted L intermediate. It also assigns an important role to the deprotonated M intermediate in the channel closure. We challenge this notion on the kinetics and suggest a red-shifted intermediate for the open channel state. In this communication we propose and test parallel conductive and nonconductive photocycles for the reaction mechanism. The spectral forms present in the reactions are partitioned between the two cycles and are assigned to intermediate states in the cycles. We reject the concept of reversibility between conductive and nonconductive states in the same reaction chain and conclude that the fast- and slow-decaying channel current components correspond to two independent conductive O1 and O2 states. We show that the WT protein, the A75E and the low-conductance D234N and S97E mutants all follow a common general mechanism, but their kinetics differ in the extent to which the conductive and nonconductive cycles contribute to it. Contrary to earlier suggestions, the M intermediate has no significant role in channelrhodopsin function. The kinetic mechanism of *Gt*ACR1 channelrhodopsin is markedly different from the proton pump BR mechanism. It follows parallel conductive and nonconductive photocycles designed to perform channel function.

## Author contributions

I.S. carried out data analysis and is the primary manuscript writer. D.S.K. prepared the manuscript.
